# Supplementary material for: Variable impact of geochemical gradients on the functional potential of bacteria, archaea, and phages from the permanently stratified Lac Pavin
Source: Microbiome. 2023 Jan 25;11:14. doi: 10.1186/s40168-022-01416-7 (PMC9875498; doi:10.1186/s40168-022-01416-7)
Supplement: Supplementary file 2 — Additional file 1: Figure S1. Protein tree describing the evolutionary relationships between putative methane monooxygenases from Lac Pavin microbial genomes and reference sequences from the broader copper membrane monooxygenase superfamily (Diamond et al. 2022). Abbreviations: AMO, ammonia monooxygenase; PMO, particulate methane monooxygenase; MMO, methane monooxygenase; HMO, hydrocarbon monooxygenase. Figure S2. Genetic potential for select carbon metabolism across compartments Lac Pavin by phylogeny. a) Relative abundance of genomes encoding various pathways across metagenomic samples. Bubble size indicates the combined coverage of all organisms of a given phylum-level designation that encode a given pathway in each sample, normalized by the total coverage of all genomes in the quality-filtered, non-redundant set. This percent coverage is averaged across all samples within a lake compartment. Figure S3. Proteome content in the CPR bacteria by depth a) Heatmap describing the presence/absence of ~1,500 protein families (≥5 sequence members) across 106 genomes of CPR bacteria. Darker patches indicate presence, while lighter patches indicate absence. Protein families are hierarchically clustered by their distribution pattern across genomes. CPR genomes are organized by their distribution across the lake water column and sediments. In panel at right, darker patches signify that a given genome was present/detectable in a given compartment, whereas a lighter patch indicates absence/below detection threshold. Dashed lines distinguish sets of genome with similar distribution patterns, e.g. the bottom represents CPR bacteria detected only in the sediments. [file 40168_2022_1416_MOESM1_ESM.pdf]

SUPPLEMENTARY FIGURES

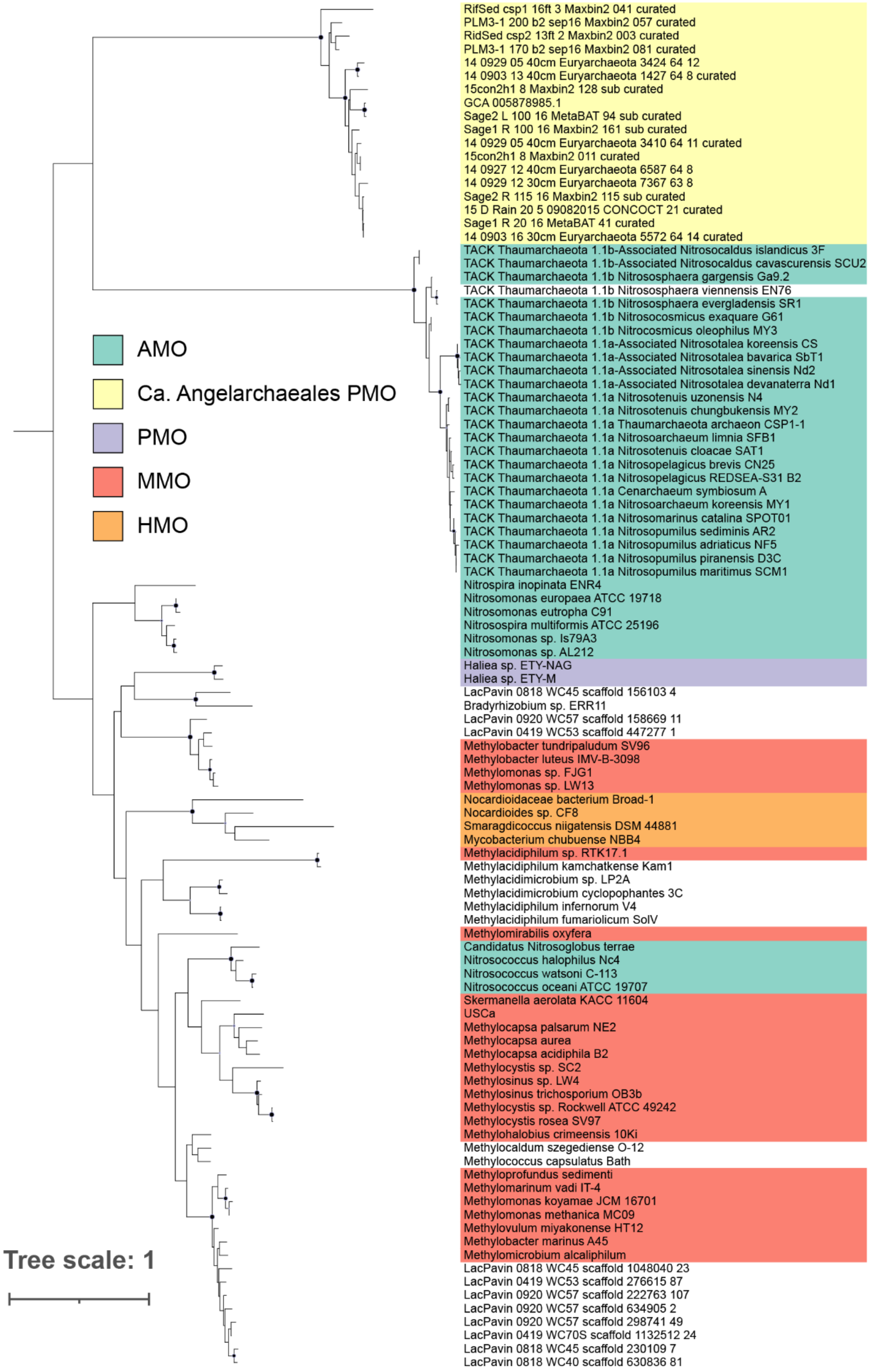

Figure S1. Protein tree describing the evolutionary relationships between putative methane monooxygenases from Lac Pavin microbial genomes and reference sequences from the broader copper membrane monooxygenase superfamily (Diamond et al. 2022). Abbreviations: AMO, ammonia monooxygenase; PMO, particulate methane monooxygenase; MMO, methane monooxygenase; HMO, hydrocarbon monooxygenase.

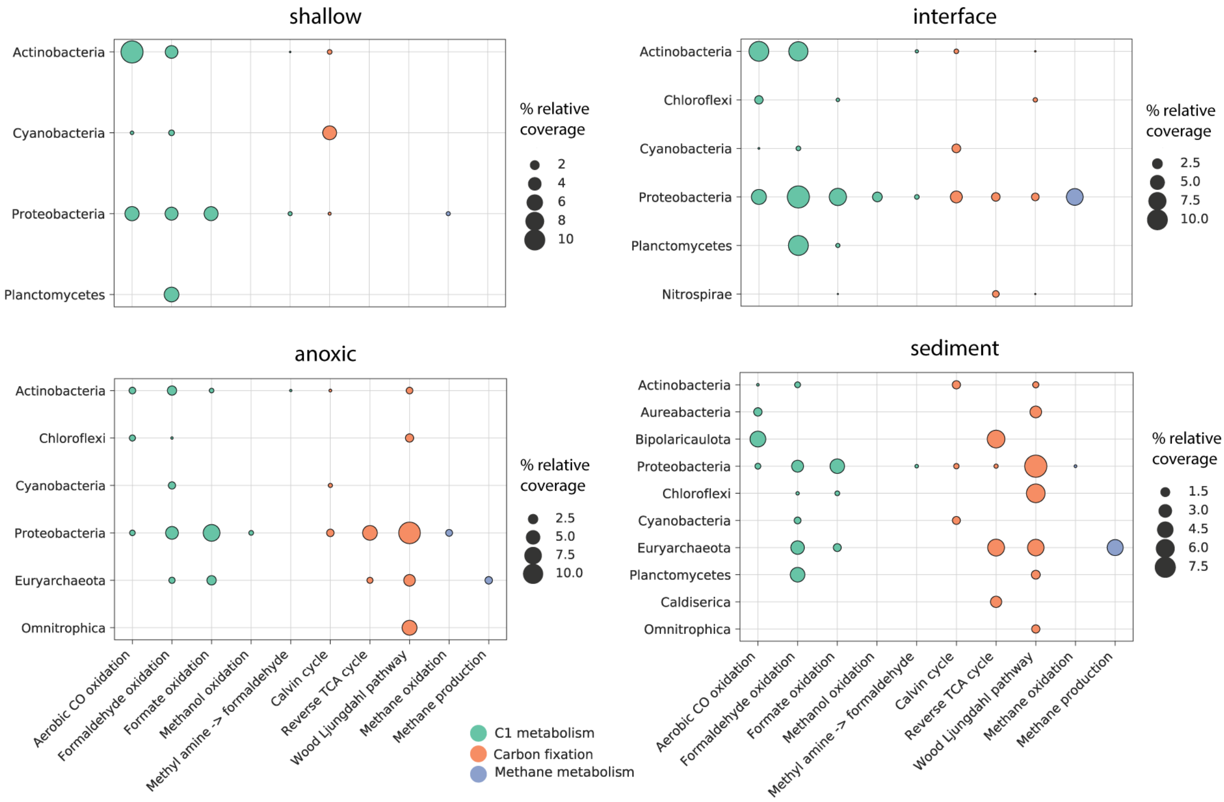

Figure S2. Genetic potential for select carbon metabolism across compartments Lac Pavin by phylogeny. **a)** Relative abundance of genomes encoding various pathways across metagenomic samples. Bubble size indicates the combined coverage of all organisms of a given phylum-level designation that encode a given pathway in each sample, normalized by the total coverage of all genomes in the quality-filtered, non-redundant set. This percent coverage is averaged across all samples within a lake compartment.

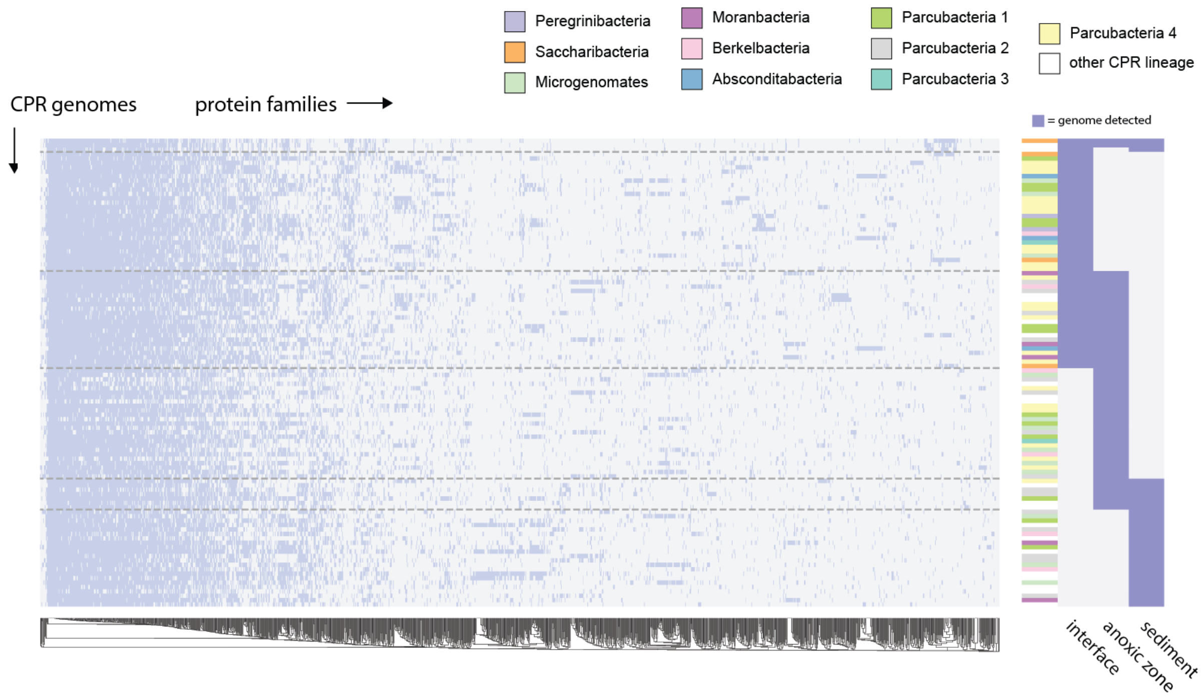

Figure S3. Proteome content in the CPR bacteria by depth **a)** Heatmap describing the presence/absence of ~1,500 protein families (≥5 sequence members) across 106 genomes of CPR bacteria. Darker patches indicate presence, while lighter patches indicate absence. Protein families are hierarchically clustered by their distribution pattern across genomes. CPR genomes are organized by their distribution across the lake water column and sediments. In panel at right, darker patches signify that a given genome was present/detectable in a given compartment, whereas a lighter patch indicates absence/below detection threshold. Dashed lines distinguish sets of genome with similar distribution patterns, e.g. the bottom represents CPR bacteria detected only in the sediments.
